# More than just a cut: a qualitative study of penile practices and their relationship to masculinity, sexuality and contagion and their implications for HIV prevention in Papua New Guinea

**DOI:** 10.1186/1472-698X-12-10

**Published:** 2012-07-20

**Authors:** Angela Kelly, Martha Kupul, Richard Nake Trumb, Herick Aeno, James Neo, Lisa Fitzgerald, Peter S Hill, John M Kaldor, Peter Siba, Andrew Vallely

**Affiliations:** 1 Sexual and Reproductive Health Unit, Papua New Guinea Institute of Medical Research, Goroka, Papua New Guinea; 2 International HIV Research Group, School of Public Health and Community Medicine, University of New South Wales, Sydney, Australia; 3 School of Population Health, University of Queensland, Brisbane, Australia; 4 Kirby Institute, University of New South Wales, Sydney, Australia

**Keywords:** HIV, Papua New Guinea, Male circumcision, Penile practices, Masculinity, Sexuality, Contagion, Cultural meaning

## Abstract

**Background:**

Male circumcision (MC) has been shown to reduce vaginal transmission of HIV to men. While community acceptability is important in a countries preparedness to introduce MC, it is equally important to map contemporary MC and other penile cutting practices, and the socio-cultural dimensions underpinning these practices.

**Methods:**

A total of 482 men and women (n = 276 and n = 210, respectively) participated in 82 semi-structured and 45 focus group discussions from four different provinces of Papua New Guinea (PNG), each representing one of the four socially and geographically diverse regions of the country.

**Results:**

Of the men interviewed 131 self-reported that they had undergone a penile alteration with some reporting multiple types. Practices were diverse and could be grouped into five broad categories: traditional (customary) penile cutting; contemporary penile cutting; medical circumcision; penile inserts; and penile bloodletting practices in which sharp objects are used to incise the glans and or inserted and withdrawn from the male urethra or in order to induce bleeding. Socio-cultural traditions, enhanced sexual pleasure and improved genital hygiene were key motivators for all forms of penile practices.

**Conclusions:**

The findings from this study highlight the complex and diverse nature of penile practices in PNG and their association with notions of masculinity, sexuality and contagion. Contemporary penile practices are critical to a community’s acceptance of MC and of a country’s ability to successfully implement MC in the context of a rich and dynamic culture of penile practices. If a MC program were to be successfully rolled out in PNG to prevent HIV it would need to work within and build upon these diverse cultural meanings and motivators for penile practices already commonly performed in PNG by men.

## Background

The penis and its foreskin have long held the interest of historians, anthropologists and the medical fraternity. Foreskins are cultural facts writes Boon [1]. In recent years they have come under far closer scrutiny as a result of three large-scale phase III clinical trials in Africa which have shown that adult male circumcision [MC] has a protective efficacy of around 60% in preventing HIV acquisition in men through vaginal intercourse [2-4]. These trials confirmed earlier observational and ecological studies on the protective nature of MC [5,6]. The success of these trials lead the World Health Organization (WHO) and UNAIDS in a joint statement in March 2007 to recommend MC as a biomedical intervention to be considered an essential component of comprehensive HIV prevention in high prevalence settings [7]. Mathematical modelling by several independent groups has shown that MC, even with partial uptake, would be highly cost-effective and could avert up to 5.7 million new HIV seroconversions and 3 million deaths over 20 years in sub-Saharan Africa [2,8,9]. The greatest impact of MC on HIV prevention is likely to be observed in communities where HIV rates are high and MC rates are low, such as in many countries in East and Southern Africa [8,10]. Targeting so called ‘core groups’ of men most at-risk of HIV acquisition is also likely to be highly effective in some settings [9,11].

While the results from these trials reinforce the penis and its foreskin as a biomedical fact, the cultural interpretation and implications of these results remain significant for HIV prevention. As a result of increased emphasis being placed on MC as a biomedical prevention in high prevalence settings research priorities has now been placed on acceptability studies of MC. Researchers such as Dowsett and Couch [12], Aggleton [13] and Hankins [14] have however argued for a broader discourse that takes account of long-term social and cultural understandings and the potential adverse as well as positive impacts of introducing MC as a population-level health intervention in non-traditional male circumcising contexts. Peltzer et al. [15] proposed a social science research agenda on MC for HIV prevention in Africa that includes the contemporary mapping of MC and/or other penile cutting practices; where and how such practices take place; the types and methods of penile cutting and circumcision practices (in non-clinical and clinical settings); the age at which such practices are carried out; their associations with male rites of passage; the religious, spiritual, social, biomedical, ascetic, and cultural dimensions; and their relationship to prevailing cultural constructions of gender and sexuality. While it may be culturally interesting to map these practices a far greater reason drives this need and that is to understand current penile practices in order to effectively communicate about MC, including its difference from other cuts, and the benefits and risks associated with MC within the context of prevailing penile cutting and modification practices, to help inform harm reduction programs and possibly to inform culturally appropriate ways of rolling out MC. Within the context of Papua New Guinea this paper is a response to such a call to document and map penile cutting practices today in order to inform public health policies, programmes and health education.

Outside of Thailand, Papua New Guinea (PNG) has the highest HIV prevalence in the Asia-Pacific region where it is estimated that 0.9 per cent of adults (15–49 years) are HIV-positive. This figure has recently revised downwards from previous estimates over 2 per cent and fears of the potential for a Sub-Saharan African like epidemic [16]. Prevalence rates are unevenly distributed through the four regions of PNG with the highest HIV prevalence in the Highlands and Southern Regions (1.02% and 1.17%, respectively) with lower estimates in the Momase and New Guinea Islands Regions (0.63% and 0.61%, respectively) [16]. While the epidemic in PNG is primarily linked to heterosexual transmission [17,18] and the national population estimate is now below 1 per cent, considerably higher rates of HIV are found amongst more at risk populations such as people who sell sex. A 2010 bio-behavioural survey in Port Moresby [19] found that 17% of the sex worker population were HIV-positive. PNG is urgently trying to find alternative means of stemming the incidence of HIV.

There is a long and well-documented history of traditional penile cutting in PNG, which is now largely out of date [20-25]. With the spread of HIV in PNG and the observational data from Africa, which indicated that MC could be protective against HIV, researchers again turned their interest to penile cutting and associated practices in PNG [24,26-31]. That said, the evidence has been dominated by behavioural data with limited sociocultural documentation and examination of the practices. Therefore, although receiving increasing attention there is still limited or at least only a partial understanding of contemporary penile cutting and associated practices and their implications for HIV prevention. It is acknowledged in the available literature that full MC (i.e. the complete removal of the foreskin) is generally uncommon in PNG, unless carried out by a medical practitioner, and that most ethnic groups do not traditionally practice penile incision [20,32,33]. Many customary penile cutting practices and initiation rituals appear to have been discontinued over the last few decades [34], but some appear to have persisted or been revived, adapted and re-interpreted [25,35]. Kempf [35] describes the appropriation of western MC practices into PNG culture and how the Ngaing of Madang Province began incorporating penile cutting (*yong*) into their cultural practices in the 1950s, as an intersection of masculinity and Christianity. In a procedure which Kempf describes as *super incision,* ‘…the foreskin is severed dorsally in a longitudinal pass of the razorblade’ as part of secret initiation rites for male adolescents. In this context ritual penile cutting is seen as purification of men’s bodies by removing maternal blood; a means of protection against sexual transmitted diseases; and a re-interpretation of the crucifixion of Jesus Christ. Hogbin [20] describes penile bloodletting practices observed among men in Wogeo Island, located near the mouth of the Sepik River, and the function that these practices served as purification rituals within prevailing religious and cultural identities in this part of North coastal PNG in the 1930s and 1940s [20]. While Tuzin [21] details penile incision practices as part of the Tambaran he also described other contexts for the use of penile bloodletting, including marriage. Below Tuzin [21] vividly described this ritualised practice of bloodletting:

After stimulating an erection he inserted a green stem about eight centimetres long into his urethra and, with a shudder that ran visibly through his thighs and torso, yanked it out. Blood oozed from the urethral opening; within three or four repeats of this action, it was spurting in a fine spray to a distance of about two metres. The stem, as I had been shown beforehand, was covered with small barbs pointing slightly downward along its length. This meant that while the stem could be easily inserted, on pulling it out the barbs caught and tore at the urethral lining. pp75.

Amongst the Hua men of the Eastern Highlands bloodletting practices of a different type were practiced, but with the same intention for purity and health. Rather than releasing blood from the penis these men engaged in bloodletting practices from the stomach, naval, lower back and bottom [36].

More recently there has been acknowledgement of the spread of non-traditional types of penile cutting, penile inserts and other practices in PNG [32,35,37-39] Hull and Budiharsana [32] describe how the use of penile inserts (including the insertion of ball bearings, beads and other objects, and the injection of silicon) is spreading among men in SE Asia and Melanesia. Some practices appear more common among certain population groups, such as prisoners.

In the 2006 PNG National HIV/AIDS Behavioural Surveillance Survey (BSS), 1358 working adult males were interviewed [26] and some form of penile cutting or circumcision was reported by 26% of truck drivers, 45% of Ramu sugar workers, 67% of military personnel, and 70% of port workers interviewed. Among 1701 out of school youth in the National Capital District, 58% of sexually active men reported that they had been ‘circumcised’ (i.e. undergone some form of penile cutting) compared to 11% among non‐sexually active men. Although this BSS did not categorise self-reports into traditional, contemporary or medical forms of penile cutting, a more recent study [31], suggests that dorsal slits are the most common form of penile cutting. For example, 25.8% (77/299) of male plantation workers reported having a dorsal slit while only 3.4% (10/298) reported full MC and 1.3% (4/299) reported having undergone a penile insert. In a study conducted among attendees at an STI clinic in Lae, Morobe Province [28], a fifth of men reported having undergone full MC (20.3%) while over 40% reported having a dorsal slit and 16% reported a penile insert. Unpublished data from the most recent integrated bio-behavioural survey of sex workers in Port Moresby [19,40] found that of the males who sold sex 55.26% reported having undergone a penile cut of some form with proportionally fewer self identifying transgender than men reported being cut (46.4% vs 60.4% respectively). Of those that reported a cut slightly more than half (52.4%) reported a dorsal slit or other cut that was not round and did not result in full foreskin removal.

The Male Circumcision Acceptability and Impact Study (MCAIS) collaborative research team undertook a qualitative study in four diverse geographical and socio-cultural settings in PNG from January – June 2009 to map current penile cutting and insert practices; to investigate and define socio-cultural norms, attitudes and beliefs relating to male and female sexuality, sexual behaviour and the broader context within which MC and related practices are being conducted; and to investigate the acceptability of medical circumcision for HIV prevention among men and women in selected locations [41,42]. The MCAIS team conducted a modified Delphi study with sexual health specialists to document and classify variants of penile cutting [38] and while there are some similarities in categories, the results in this study are produced from a different data set but should be viewed as part of this papers wider program of research. In this article we present the key findings on current penile cutting practices from a qualitative study of communities throughout PNG in order to contextualise any future discussion of MC within the current cultural context of penile practices and associated notions of masculinity.

## Methods

**Figure 1:**
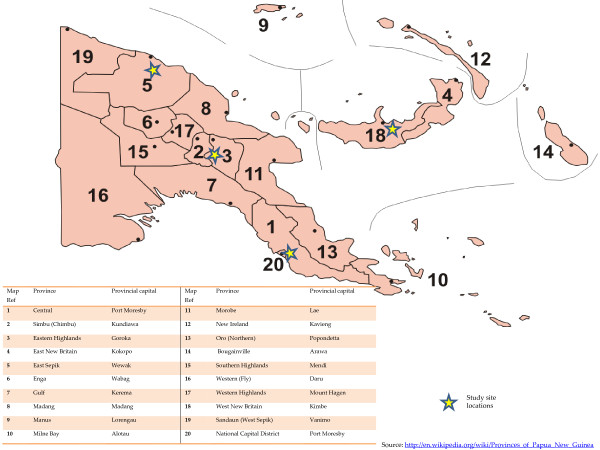
Map of Papua New Guinea and study locations.

A multi-method qualitative research study was undertaken in four provinces, each representing one of the four socially and geographically diverse regions of the country: National Capital District (NCD of the Southern Region); Eastern Highlands Province (EHP of the Highlands Region); East Sepik Province (ESP of the Momase Region); and West New Britain Province (WNB of the New Guinea Islands) (Figure 1). These sites were selected during a National Stakeholders Workshop held in Port Moresby in May 2008, attended by in-country government and non-government stakeholders including representatives from the National Department of Health, Save the Children in PNG and HOPE Worldwide; and researchers from the PNG IMR and Australia-based research team. Approval from key local authorities, chiefs and/or other stakeholders was obtained prior to the arrival of the research team in each study location. Researchers conducted an interactive community meeting (*tok save*) at each proposed research site prior to the start of the study in that location. The National Capital District was selected because it is the capital of PNG and has attracted in-migration from all regions of the country and beyond creating a diversity of peoples and practices. Eastern Highlands Province is considered a non-traditional penile cutting cultural area but is linked with major areas of the country through the Highlands Highway. East Sepik Province and West New Britain Province have anthropologically documented traditional penile cutting practices [20,22,23,25,43]. In each of these provinces the research team conducted research amongst multiple cultural and language groups, therefore we do not provide the anthropological details of each.

An iterative, purposive sampling technique was used to identify potential study participants following initial contact and interviews with key local stakeholders and community members at each study location. A form of purposive sampling known as snowball sampling was used to gain access to men who had undergone penile cutting, inserts or associated practices. Other participants were recruited through schools, universities, workplaces, villages and church groups. The research team actively sought to recruit participants with unique insights and lived experiences into the area study, such as men performing penile cutting or other practices in the community; staff from the National, Provincial and District AIDS Councils, ex-prison inmates; village chiefs and elders with a knowledge of traditional cultural practices e.g. associated with male initiation ceremonies.

A combination of qualitative research methods was used in this study: focus group discussions and semi-structured interviews. A total of 482 men and women participated in 82 semi-structured interviews and 45 focus group discussions in four study locations. 24 focus group discussions were conducted with men and 21 with women. 65 men participated in semi-structured interviews while 16 women did. (Table 1) In total, of the 486 participants, 276 were men and 210 women. Data saturation was reached in relation to both research site and gender.

**Table 1:** Interview type by study location

	**Study locations**				**Gender**	
**Type of interview**	**EHP**	**ESP**	**WNB**	**NCD**	**Men**	**Women**
**Focus group discussions**	8	10	14	13	24	21
**In-depth interviews**	11	31	17	24	65	18

All focus group discussions and semi-structured interviews were digitally recorded, transcribed verbatim and where necessary translated from *Tok Pisin* (a lingua franca of PNG) to English and thematically analyzed by a team of five researchers. A codebook was developed and all interviews’ were double coded. In cases of discrepancy in coding a third researcher coded the selected text in question. All identifiable information was removed, pseudonyms given to each participant and names of village or settlement removed.

As a qualitative study the primary limitation was the use of self-reported penile modification. In the absence of a clinical examination participants’ self-reports could not be validated.

All participants provided written informed consent in either English or TokPisin. All participants were advised that they could withdraw from the study at any time without prejudice.

Ethical approval was obtained from the PNG IMR Institutional Review Board, the Medical Research Advisory Committee in PNG and the Research Advisory Committee of the PNG National AIDS Council as well as from the Human Research Ethics Committees of the University of Queensland and the University of New South Wales in Australia.

## Results

Of the male participants (N = 276), 131 identified as having conducted some form of penile practice. Some men reported having undergoing several penile modification practices, for example both penile bloodletting and a contemporary cut. Of these 131 men, 150 practices were reported: 51 men had a traditional penile cut; 70 a contemporary penile cut; 6 had undergone medical circumcision; 10 had inserted foreign objects into the foreskin and/or dorsal penile shaft; and 12 men had conducted penile bloodletting practices.

As a result of the qualitative findings from this study, penile practices in study communities could be grouped into five broad categories: (Table 2)

1. Traditional (customary) penile cutting: associated with male initiation ceremonies and conducted with the support and involvement of men and women form the local community;

2. Contemporary penile cutting: conducted without associated ceremonial practices and in the absence of customary community support or engagement;

3. Medical circumcision: carried out by trained health workers at designated health facilities;

4. Penile inserts: the insertion of foreign objects into the foreskin, foreskin remnant [following traditional or contemporary penile cutting or medical circumcision] or into the skin of the penile shaft;

5. Penile bloodletting: in which either sharp objects are incised or rubbed on the glans to induce bleeding or objects are inserted and withdrawn from the male urethra to induce bleeding.

**Table 2:** Summary of categories of penile modification practices identified in this study

**1**	**Traditional (customary) penile cutting**
**2**	**Contemporary penile cutting**
**3**	**Medical circumcision**
**4**	**Penile inserts**
**5**	**Penile bloodletting**

No men in our study reported that they had participated in any form of penile injection (the injection of silicon oil or other products into the skin of the penile shaft and/or foreskin) [29,31].

### Traditional penile cutting

#### Context

Key informants in ESP and WNB identified local communities in which they believed traditional penile cutting practices and male initiation ceremonies continue to take place. In ESP, subsequent community consultation and qualitative inquiry revealed that such practices had now largely been discontinued in study communities and where penile cutting still existed, had been appropriated and removed from the traditional initiation context. In WNB, traditional forms of penile cutting and associated initiation rites remained important cultural practices in the communities visited, with all men expected to undergo the custom.

‘No men in [village named] will avoid custom. No one. All must go through the process of custom [traditional penile cutting]. Children must still go through custom. No men will avoid it. All still have to go through custom. *Desmond, M, WNB.*

The expectation to be initiated was evident not only from informants in WNB but also from respondents residing in Port Moresby but whom originated from WNB. Such informants who were already initiated prior to moving to the capital spoke of their cultural obligation to return home in order for their male children to undergo initiation.

In WNB, penile cutting is primarily the responsibility of older (initiated) males in the community. These men take charge of all aspects of decision-making and the order of ceremony. These same meet in the *haus man* or *haus boi* (a house traditionally designated for the exclusive use of men) and decide upon who the next group of novices are to undergo initiation, when and how the ceremony (which includes feasting and celebrations) is to be conducted.^a^ Women and men in the community are then instructed to start preparing for the occasion by making food gardens, obtaining and saving money, raising or buying pigs to be eaten or presented during the ceremony.

During the ceremony, it is only the men who are allowed to witness the actual cutting of the penis. At this time of cutting male ancestral spirits (*tumbuan* in the local language) are present, mixing and dancing with the men and important cultural knowledge is passed from men to boys, including the involvement of spiritual knowledge and practices. These are closely held secrets and cannot be shared with others. According to one respondent interviewed in Port Moresby but who originated from WNB,^b^ during this first stage of initiation members of his clan learn who their ancestral spirit is; this spirit represents the clan’s origin. Learning about this spirit involves coming to understand ‘our origin, our clan’s status and the land and the forest and of the seas, for example which reef we should go for fishing and that kind of thing. So it involves who we are and what we have as a clan.’ (*Andrew, M, NCD*)*.*

While the men take charge of customary practices, women’s involvement in the initiation of young males is restricted to singing and dancing (*samsam* in the local language), and the distribution of food and gifts. Female respondents in several study communities were however found to be highly knowledgeable about the overall customs and practices regarding traditional penile cutting and male initiation, and able to explain in detail the process of initial preparations, how penile cutting is performed, and the procedures and customs regarding the disposal of blood. The restriction of women (and any uninitiated male) from the actual moment of cutting and its associated ritual is not a sign of antagonism and separation as it may be read from early anthropological work. Rather, women appear to play what Stewart and Strathern [44] would describe as a complimentary and co-operative role as evident by the descriptions provided by the informants.

Initiation typically takes place when men are in their late teens or early twenties: ‘When we become young adults …they cut us’ (*Elgar, M, WNB).* However, there were cultural groups that reported initiating their males in childhood or early puberty. In one village when talking with a group of women, one of the mothers (with her husband’s approval) showed the female researchers her young boys’ penile cuts. These boys were aged between 4 and 6 years. It was confirmed by another informant in this village, a father of a boy recently initiated, that this was indeed a typical age to traditionally cut boys in this community.

#### Customary obligations to family

In WNB study communities, traditional penile practices were considered within the context of a wider cultural framework whose observation enables customary obligations towards family members, in-laws and other relatives to be fulfilled through the exchange of goods and in-kind contributions. The parents of prospective initiates pay those who perform penile cutting (incisors as Tuzin calls them) with food, pigs and money. These payments form an integral part of the initiation ceremony. They signify the parents’ appreciation, and act as a type of compensation, of the cutter for handling their son’s blood. Such blood is viewed as contaminated thus the need for compensation. This process was described by Howard whose son was recently initiated:

It depends on how many children we put inside [the *haus boi*]. If five children, then it’s five men. One to a child. And if a child is frightened or such, it will be one shot. If one cuts here and the other cuts there, it will be quicker and will end. We will also prepare to give food to them regarding this. If there are no pigs, we will buy chickens and lamb flaps from the store and we will *mumu* [cook in traditional earth oven] the food and give them to whoever does this process. *Howard, M, WNB*.

These activities also enable broader traditional obligations to be met, such as strengthening family, cultural and social ties within communities. One commonly mentioned customary practice is for the parents of initiates to make payments in food, money and other valuables to the maternal uncles of the boy being cut as compensation for the loss of blood by the boy. This familial and cultural obligation was described by Yolanda, a woman whose community till practices traditional penile cutting:

When we cut the foreskins of our children, we give ‘Bride Price’ to the uncles of the child that we have cut. We don’t just cut. There will be a payment to the uncles from the mother’s side and then we cut. They get this payment for the spilling of blood by the child. *Yolanda, W, WNB*.

Although not described in this paper, this cultural and familial obligation is central to the debate around acceptability of MC for women from WNB where traditional penile cutting continues [41]. It is the women’s family (brothers) not the fathers, who are compensated in this manner.

#### Stigma

In communities where there is traditional penile cutting, social differences exist between those who have and those who have not been cut. This power differential is very important with those who have not been initiated (and therefore cut) are stigmatized, ridiculed and mocked as a result of their exclusion from traditional knowledge [45]. Both men and women referred to this form of cultural discrimination:

It’s like this; to be cut is for people to be happy for you. They won’t mock you. If you are not cut, they will make fun of you, like you still have your foreskin, you know, people will make fun of you. So, when you are cut, people will be happy for you. They will be happy for you and when they see you, they will say you are a man and they won’t make fun of you. *Ismael, M, WNB*.

People in [village] name men with foreskins *utilusa* meaning foreskin; *aipu* is also used. It is the same for us for those who have not cut yet. When they have cut, they won’t use these names on them. They will make fun of those not cut yet. It’s an expression. *Desmond, M, WNB*.

In [our language], those that are not cut are called *kapu* or *kapundoluur* [with foreskin]. *Keyna, F, WNB*.

Discussions with study participants suggest that these names may have a broader resonance and serve to denote or imply lower status within these societies. It also denotes those who are not yet men. It is understood that the fathers and the families of the boys and men who are uncut lack customary and modern forms of wealth, such as money, pigs and *tambu* (shells) required to undertake such an expensive activity, and are therefore looked down upon by others in the community:

If a man doesn’t do custom [traditional penile cutting] for his son, he will feel really bad and ashamed. He will feel ashamed towards his peers who have done custom to their sons and he hasn’t. People will say, ‘That man is poor, he has no *tambu*, no money’. *Xenia, F, WNB*.

#### Masculinity

It was evident that men in societies that practice traditional penile cutting were conscious of how women view and accept them as men in their societies, and that women are said to prefer men who have been cut for marriage and as sexual partners:

If I’m not cut yet, one thing we fear is that if I ask a women, especially from [named village], she will say “I don’t want you because you have not cut your penis yet”. That is what has made us to cut. Once I am cut, then I will become motivated to do it [sex]. *Garinga, M, WNB*.

But it is not only the views of women that are important in the transformation of boys into men. The whole purpose of these initiation rites, reflects Tuzin [34] to make boys men and the men’s house is itself a ‘masculine sanctuary’ (p.64). Tuzin writes that with the Revival movement a repeal of customary practices associated with the Tambaran occurred and that this with the revelation of the secrets of the Tambaran resulted in the ‘murder of masculinity’ (p.65) thus reemphasizing the relationship between this practice, the social place where it occurs and the creation of masculine identity [34]. Therefore, as Herdt [46] wrote, ‘[t]he uninitiated boy is problematic. He is male but not biologically, socially or psychologically masculine’ (p.97).

In addition to perceived masculinity, social status, personal and family wealth, the apparent preference among women in such communities for men who have undergone penile cutting also appears motivated by beliefs around genital hygiene, cleanliness and their potential impact on fertility:

We the women from here don’t like men who have not cut their penis foreskin. There is a lot of dirt in them. We like the men from here who have already been cut…those men who have not cut their penis; there is a lot of dirt in them. When to get together with women and their water [semen] come into us, we are getting dirty water into us and we will become sick from it. When our men are circumcised, their water is clean and will go straight to the us, the women so we can have babies. *Melania, F, WNB*.

#### Types of cuts

**Figure 2:**
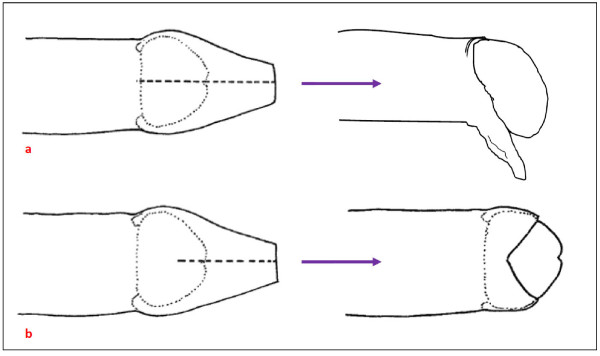
**Diagrams of the most common penile cut. a:** longitudinal dorsal slit, complete: resulting in lateral retraction of the foreskin and a flap of skin that hangs down below the penis;. **b:** longitudinal dorsal slit, incomplete: resulting in ‘V’ shaped opening legend text.

The most common type of traditional cut reported by participants in WNB was a longitudinal dorsal slit of the foreskin, referred to locally as the ‘straight cut’ or ‘long cut’ (Figure 2a). This appears analogous to what Kempf [35] described as ‘super incision’. In this procedure, respondents advised that the foreskin is pulled over the head of the penis and a straight slit is cut which ends at the opening of foreskin adjacent to the tip of the glans. In this type of cut the foreskin is not removed but left to hang under the penis or rolled back to the base of the penis. Another form of traditional cut reported was an incomplete version of the ‘long cut’ in which the foreskin is only partially dissected, leaving a ‘V’ shape at the distal edge of the foreskin which widens as the young male grows (Figure 2b) and which is commonly referred to as a ‘V cut’.

#### Cutting procedures, practices and care

Historically, blades of bamboo were used to cut the penis. Amongst the people of Ilahita of the East Sepik Province this bamboo razor is known as *falanga* and is created from a thorny palm from a sago plant. Modernity has made razor blades and surgical blades readily available, and these are now the main instruments used. After cutting, the initiate is placed beside a fire that is thought to help the wound stop bleeding. In some cases initiates are taken to the sea to be bathed immediately after cutting to prevent infection and aid wound healing.

Some respondents described the use of specific herbs that are put on the wound to stop bleeding and to facilitate the healing process. Respondents advised that the use of such herbs has become less frequent in recent years and those modern medicines and dressings for wounds are now more commonly used:

During traditional times, we have our own medicines. When we don’t have medicine, we are not sure but we see what older men do with coconut, like with the shoots of the coconut tree which has some medicine in the shoots. This is a strong medicine for us in the village and we use it on the new cuts…This we use in the village but now many other medicines are available and we are using amoxy [amoxicillin] to drink …when we use this we saw that the white man’s medicine is better than our traditional ones. *Garinger, M, WNB*.

Before and after penile cutting is carried out, those to be initiated, their family and peers are required to observe certain customary practices considered necessary to avoid complications such as pain, infections and excessive bleeding. For example, Bike from WNB described the following instructions:

I tell them, “When I cut you, don’t drink a lot of water. Drink a little only. If you drink a lot of water, the sore will become swollen. When you want to drink, have only a little to soothe your throat”. *Bike, M, WNB*.

Another frequently mentioned sanction was avoidance of any form of sexual contact prior to initiation. Avoidance was said to prevent complications during cutting and the subsequent healing process but also appeared to reflect more fundamental, deeply-held concerns regarding the risks of initiates being contaminated by ‘the smell of women’:

Those that have not been cut yet, there is a serious restriction on them. Do not be near women… there is a taboo on them because it will affect you during the time for custom. You will have big problems. The smell of women will go into you and when you go into the way of custom, the elders have already made magic and such things on the initiates. You have to stay fresh, you body must be okay and you can go into the ways of custom. This is a law of the house man. ^c^*Desmond, M, WNB*.

### Contemporary forms of penile cutting

#### Context

Contemporary penile cutting was reported in all study locations. Feasting, community participation and other customary observations required in traditional cutting and male initiation ceremonies were not associated with contemporary penile cutting practices in any of the four study sites. Many respondents believed that contemporary cutting was introduced by ‘outsiders’ or ‘foreigners’, although they were often unable or unwilling to specify or to articulate this belief more fully. Respondents in some communities referred to Papua New Guinean men entering the community anew or returning to their community having lived in another area within PNG for some time; others referred to the influence of people from Asia (‘Asians’) and other overseas visitors. This highlights the important role that both modernity and development are playing in contemporary penile practices in PNG.

Contemporary cutting is carried out in a variety of settings including rural villages, urban settlements, towns, schools and prisons, and typically in locations adjacent to water in order to minimize blood loss, allow fresh cuts to be cleaned and to facilitate the disposal of blood (e.g. private houses, secluded bush areas beside the sea or river).

Ex-prisoners and Correctional Service Officers confirmed that contemporary penile cutting and penile inserts are widespread and commonplace in prisons, and said to have become part of prison culture. Ex-prisoners reported that prison health workers are assisting prisoners by supplying inmates with medicines and dressings. In most cases, inmates are cut in secret by other inmates without the knowledge of prison officials but may subsequently seek medical help at the prison health facility. Respondents said they had heard very little of such practices before going to prison.

All respondents who had undergone this form of penile cutting reported there being a single cutter involved, irrespective of the number of men being cut at any one time. Cutting is typically conducted by a trusted peer or an older male with experience in this procedure.

#### Motivators

Contemporary penile cutting appeared most commonly practiced among young adult males, many of whom said they had been influenced by peers. The most common specific motivating factors identified were the perceived positive health and hygiene benefits of the procedure, and the potential for increased sexual pleasure for both themselves and their female sexual partners. There did not appear to be any association with religious beliefs and with their emphasis on health. Respondents believed that cutting would prevent the accumulation of dirt and waste (*kok waste*, *kokpekpek*) under the foreskin and prevent sexually transmitted infections, including HIV (fatal disease):

I see that it is good I cut my foreskin because when it is there, I felt that it is itchy. *Lekemi, M, NCD*.

The people say that when you still have the skin and you do all these bad activities, have sex and all this, and if some dirt and rubbish stay stuck on your cock, you can get sick with diseases like gonorrhoea and syphilis…the skin is the thing that brings all this kind of thing to create sick. When you remove the skin and stay, you will not face all this kind of things. *Camilus, M, ESP*.

I don’t want to catch any fatal disease that’s why I had to do it. *Naldo, M, EHP*.

In addition to hygiene, there were many others who said they believed that contemporary penile cutting increased and prolonged sexual pleasure and delayed ejaculation during vaginal sex.

Yes, they used to say that when you cut and go stay with a woman, you will have sex for a long time because when the skin is there…you won’t have sex for a long time. When you cut it, your penis will be free and you can have sex for a long time. Like you will have sex on and on. Some longer minutes… and when you have foreskin and you sleep with a woman, it will kind of like explode, like that, like you will release quickly but when you have cut it already, you can stay with a women for a long time. You stay a long time and you won’t release quickly, you will stay with her for a long time. *Isaiah, M, ESP*.

#### Types of cuts, procedures and care

**Figure 3:**
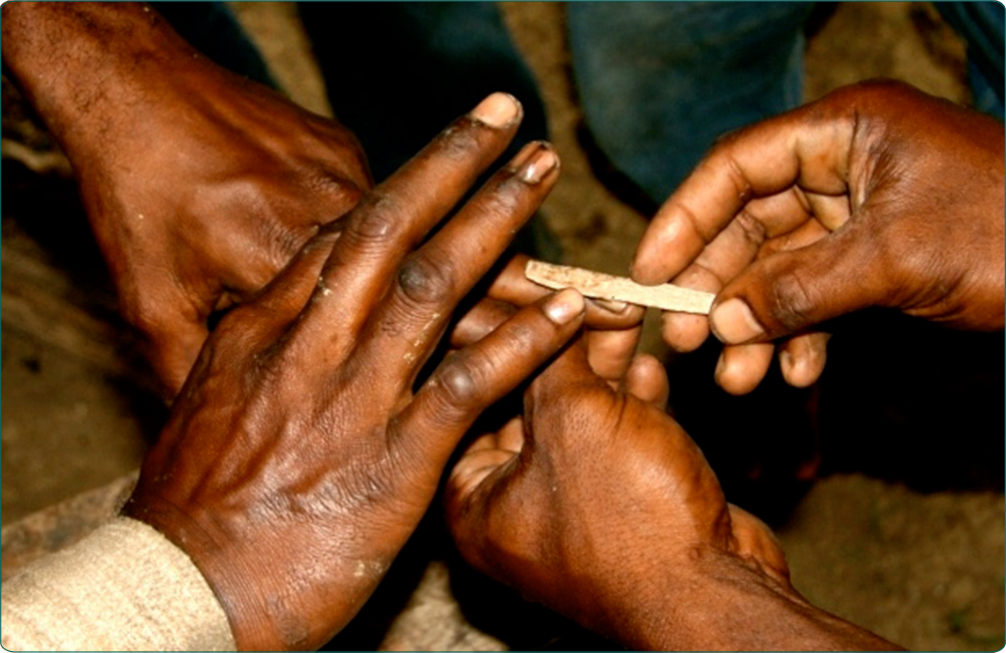
**Photograph of male participants showing how a longitudinal cut is performed**.

The most common type of contemporary cut reported was the ‘straight cut’, analogous to the longitudinal dorsal slit reported above (Figure 2a). This cut is different from that advocated in MC in a very important way. Although the foreskin changes profile and it can appear that the foreskin is removed in fact the foreskin that has undergone a dorsal slit remains. Respondents described a number of cutting procedures, the most common being to insert a flat stick (such as an ice-lolly or ‘paddle pop’ stick) between the uppermost (dorsal) foreskin and the glans penis, stretching the foreskin over the stick, then using a razor or surgical blade to slit the foreskin longitudinally (Figure 3). As in traditional forms of penile cutting, the final aesthetic result is dependent on the length of the cut.

Cutting of it, when we go they will push a thing like an ice cream [stick] or bamboo, push it into the foreskin of the penis and then they will hit it a couple of times, move the veins to the side [using their fingers] and then they will cut it. *Raymond, M, ESP*.

Other varieties of contemporary cutting, although reportedly far less common, have emerged including multiple longitudinal slits and procedures that fashion a slit in the dorsal foreskin through which the glans is then forced:

Okay, the other too is just at the tip of the foreskin. I don’t cut it to the end, I just do a slit and push the penis out [through the slit] and it stays outside. *Tumbe, M, EHP*.

Following cutting, a variety of traditional and/or modern remedies are used to control bleeding, reduce pain and to facilitate wound healing. The majority of respondents reported using modern medicines and dressings only.

I cut *setri* [bark obtained from a particular type of small tree] and scrape it. After he cut my penis, we wait until this bad blood^d^ has come out then he squeezes the *setri* juice [a greenish coloured liquid] into the cut. *Henriot, M, ESP*.

These days we use the medicine I mentioned like Dettol and Panadol. *Daniel, M, NCD.*

After I put the medicine, okay, I put cotton wool on top. I don’t cover the urine hole. I don’t hide it. *Tumbe, M, EHP*.

Medicines and dressings were often obtained through friends and relatives working at local health care facilities.

Sometimes they tell some friend or relatives who work at the hospital. They used to say “we have are going to do this and so you provide medicine to us and we will do such”. Okay, those that do not have such [friends or relatives working in hospitals] and want to do it, they used to go and say that they have sores and try to explain. When they say they have done such [penile cut], the nurses get cross at them and tell them not to do such as there are ways to circumcise men so you must come to the hospital to do such if you want it done. *Daniel, M, NCD*.

### Medical circumcision

Almost all respondents with medical circumcision were from ESP. The MC program had been promoted for HIV prevention in Wewak since late 2006. According to the coordinator of the program, who was interviewed for this study, over 490 procedures had been conducted by March 2009. Respondents’ principal motivation for medical circumcision was because they had heard from family, friends, the local media and health workers that circumcision could reduce their chances of acquiring HIV:

I was frightened about this sickness so I went to the Provincial Health Office to see [name], the person in charge of male circumcision. He asked us to be circumcised and I also agreed because I was scared of this disease [HIV]. *Waipware, M, ESP*.

I went to the hospital, you know, not only because of HIV. There are other kinds of sickness from sex. Therefore, I thought about myself and about this and I went to the hospital to be cut. *Hadrian, M, ESP*.

One participant however reported attempting to prove that medical circumcision was protective by having unprotected sex with many partners after being circumcised:

As for myself, after circumcision I wanted to try it out so I had sex with six different women. After this operation, I did not have sex with my wife but with different women and [named] did blood check on me and I was okay. So now I am a regular face to the blood bank, I go there every three months for blood check and get my results. *Waipware, M, ESP*.

Playing a more passive role as ‘patient’ in this form of penile modification meant that participants had very limited knowledge of the procedure they underwent. Besides the cut itself, this passivity in the circumcision process is an important distinction between it and other forms of penile modification.

### Penile inserts

#### Objects, places and procedures

**Figure 4:**
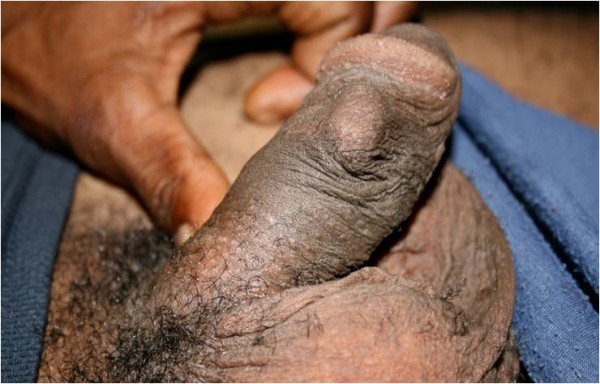
Penile insert: ball bearing in skin of penile shaft.

Penile insert practices (the insertion of foreign objects into the foreskin, foreskin remnant or into the skin of the penile shaft) were reported in all study areas. A variety of objects were reportedly used including metal ball bearings, beads (*pokpok*), toothpaste tube caps, turtle shell, horse or pig tail hair, chicken feather, string, threads and earrings (Figure 4). As described by one informant:

I used the handle of a toothbrush, which I filed until it is sharp. You know those plastic ones. I cut it and file them until they are round. I poke the skin on my penis and put them [the balls] inside. That’s all. I get some medicine and the sore dries up. *Gratian, M, WN*.

These objects appear similar to those identified by the PNG IMR during the National HIV Mapping project.^e^ Unlike penile cutting practices, respondents had not heard of a ‘cutter’ or ‘operator’ carrying out this practice on others: all inserts appeared to be inserted by the individual. Often these objects were inserted following a contemporary penile cut.

This skin, this leftover skin that they have cut and had fallen down, that they pick up. They mark it here and mark it there so the bearings can go and stay and there will be space to move around, you know. If you put them to be close together, there will be no space so when you have sex or such, there will be frictions and blisters. So it will be a waste because you will cut and put another again, you know, it is such. So mark it before and later pull the skin up and poke it and put it. *Bolton, M, NCD*.

Some respondents described inserting a number of objects, which were subsequently removed. For example, Bolton from Port Moresby described how he inserted a total of five ball bearings but later removed three. The respondent did not elaborate on why he removed three of his inserts. All respondents irrespective of gender agreed that penile inserts were not a traditional Papua New Guinean practice but rather had been imported by ‘foreigners’ (specifically ‘Asians’). In PNG there is little to no cultural distinction made between people of different Asian heritages. All people, irrespective of their place of origin are refereed to as ‘Asian’ as opposed, for example, being referred to as being Indonesian, Korean or Malaysian. Those men who had penile inserts said that they had learned of this practice from friends (Asian and Papua New Guinean), from ex-prisoners or whilst in prison themselves.

Like I worked with Asians in the timber operations and I saw lots of objects they used. They used bearings, they put necklaces inside to make the head of the penis [larger]. They made earrings and such things. They put it inside to increase their feelings. That is why they do all sorts of things to themselves. I haven’t done it but I have only seen it [penile insert]. *Jason, M, ESP*.

#### Motivators

According to men with and without penile inserts, these insert practices appeared to be primarily motivated by a belief that they would increase sexual pleasure for both the men and their female partners. Increased pleasure would be the result of an enlarged penis and the friction that the inserts would cause during sex:

Everyone, like I will say, had the same thought, to enjoy sex so they want to put this thing so sometimes they say: “My stick [penis] must be small; I will cut and put this thing so I will be outstanding”. *Bolton, M, NCD*.

This was also said to be an important way for a man ‘to keep his woman’ because she would now be sexually satisfied in a way that she had not been before:

Women will feel that the men have large penis or a small penis. They will feel that we have a large penis so they won’t leave us. *Tumbe, M, EHP*.

The opinions of these men that puts this thing, they feel that when they have sex with women, the ladies will feel like, when they have sex with them, the ladies will feel really satisfied or some kind of some high feelings on top of this…If you are a man who has this kind of bearing or this thing on your penis and you have sex with her. It will be like she will really feel it, like she got one deep, receive a very big thing or like that. *Camilus, M, ESP*.

This means that it’s feeling. If you stay [have sex] with a woman, she will not forget you. That is what it means. Every time she will come back to you. If she is a married woman, she will always think of you. *Xaivier, M, ESP*.

Although all of the respondents with a penile insert reported increased sexual pleasure as their primary motivation for carrying out insert practices, several respondents reported that part of his motivation was to punish or cause harm to women, especially those considered to be a ‘showoff’; a *pamuk meri* (woman perceived to have had multiple sexual partners or colloquially a ‘prostitute’); those who had previously rejected an invitation to have sex or to start a relationship with them; or a *4 Kopi meri.* Coffee is graded according to quality and a grade four bean is of very poor quality. When a woman is referred to as a ‘4 Kopi meri’ a man is symbolically expressing that she is of poor quality, cheap and easily disposed off. As described by men with inserts:

One way is for sex. I used to think that, “okay you woman, you are a bighead. I will put bearings and see you”. *Ismael, M, WNB*.

It is like, when they put a bearing and have sex with women, blood used to come out. They used [it] to spoil the women’s body. *Lekemi, M, ESP*.

It appears that for some of the men with penile inserts the sexual experience of inserts for women was unclear with a blurring of pleasure and pain as a man with an insert himself described:

Yeah, it’s for making the women like, those who have this behaviour of prostitution, they will feel it like painful or more pleasure compared to the ones who don’t have these bearings or animal hairs and all this. *Camilus, M, ESP*.

Drawing on from this duality of inserts, women said that the pleasure of inserts was one sided.

For the men they are thinking of themselves to satisfy their feelings but they never think about the women*. Fiona, F, ESP*.

They are only thinking about themselves and their feelings and their sexual desires *Sylwyn, F, EHP*.

Not surprisingly then, all women in the study who discussed penile inserts said that men had them inserted in order to cause vaginal pain to women, to cause bleeding and to damage the vagina, usually, but not always, to *pamuk meris* (prostitutes).

According to the women in the study two categories of sex increased the pain associated with inserts. The first was in the case of a man with an insert having sex with a virgin and the second was when men with these inserts engaged in ‘rough sex’. So concerned about the pain and damage caused by penile inserts one of the women in the study who sold sex reported that in order to protect herself from male clients with such inserts she always held the clients penis in her hand before she had intercourse. That way she could check for bearings as well as sores; she did this because ‘it’s my safety’.

In an extreme account of penile inserts a health care worker recounted an experience from Madang: ‘when the penis is erect, the person wants to rape a lady, it will tear it, the penis will actually damage the vagina, right into the cervix’ (*Helsa, F, WNB*). Responding to this case the informant said that the police captured the rapist and forcibly removed his penile inserts using pliers. Another health care worker shared her experience of tending to a woman who presented at the hospital after a traumatic rape with inserts where she said:

A lady was raped and brought in bleeding, so we inserted the speculum and we saw the cervix…. Her cervix broke right in, and then I was wondering, why and how did her cervix break right inside? Then, the sister in charge got up and said that person who raped the lady must have used a bearing. He must have inserted bearings in his penis so it really tore the lady up, so we have to suture the cervix, the posterior and interior cervix, so it stopped the bleeding. *Greta, F, WNB*.

For women there was no discrepancy about the reason for men undergoing penile inserts.

Correctional Services Officers and ex-prisoners felt that a key-motivating factor among prisoners was boredom:

In the prison, you just stay and there is nothing much to do. One way is to look for things to do to keep busy. To pass time, they do these things like putting bearings in the penis. *Ismael, M, WNB*.

All ex-prisoners interviewed had seen someone with a penile insert, performed such a procedure themselves or seen the operation being carried out. Sometimes, the insertion of these objects was the result of peer pressure:

For me, it’s like, when I was a teenager, I went to prison and there were big boys who were there and it was from their mouth; they said, and so we put it. So it wasn’t my choice. *Gratian, M, WNB*.

### Penile bloodletting

EHP, ESP and NCD participants described penile bloodletting practices. Only in one village in the ESP was the traditional practice of inserting a blade of grass into the urethra still practiced while in other areas of the ESP, EHP and NCD participants described incising the head of the penis by cutting and or rubbing of objects in order to release blood. Both traditional and contemporary bloodletting practices were described in study communities. Penile bloodletting practices shared common underlying cultural meanings, significance and motivators based on constructions of maleness, masculinity and gendered societal roles, analogous to the practices and contexts previously described by in a variety of settings in PNG [20,22,23,43].

**Figure 5:**
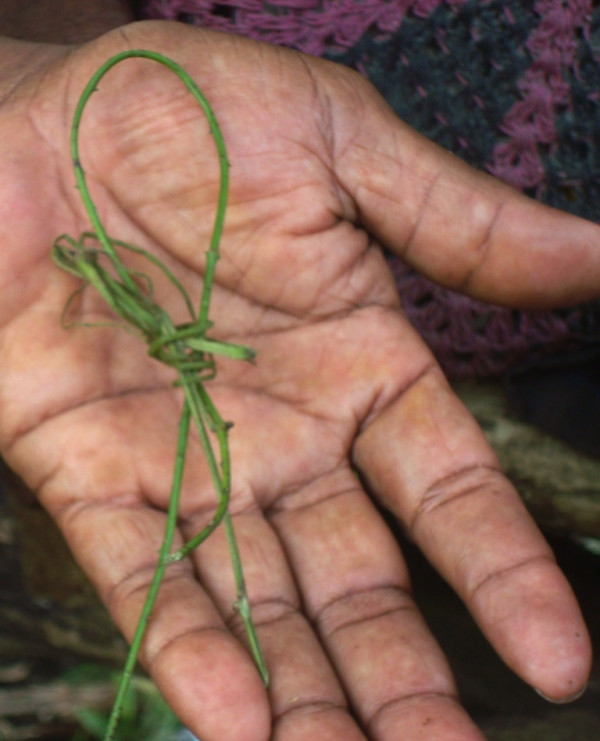
Vine used in penile bloodletting practice.

The most common objects used for penile bloodletting were a blade of grass with sharp edges or a short length of vine having minute thorns (Figure 5), that when inserted into the urethra and quickly removed result in bleeding:

Sometimes, there are two kinds but this “kunai” [spear-shaped] grass is best. It is best. It has four edges…It is straight so when they push it inside and pull it just once and it cuts the inside of the cock. It does such and the blood comes out quickly. *Sailor, M, ESP*.

Respondents in EHP described the use of a short spear (with or without a broken piece of glass from a bottle on the end) or sharp slivers of bamboo to induce penile bleeding; and the use of the bark and/or leaves from specific trees or razor blades to induce bleeding of the penile glands.

#### Motivators

The cultural significance and purpose of both traditional and contemporary forms of penile shooting were deeply embodied within notions of masculinity, male sexual and general health, and the need to purge ‘polluted’ maternal blood:

The first time we do this is because when our mother gave birth to us, this blood is in us. When we go to the men’s house, they shoot to release rubbish [blood] which our mother gave us at birth. *Jason, M, ESP*.

The natural way for girls to remove this blood, according to the men is menstruation while with boys in order to release a mother’s blood are required to induce a metaphoric equivalent of menstruation, a male menarcheal ritual:

When women have their monthly period they get rid of this waste and let it go out. For us men, this is the only way. *Sailor, M, ESP*.

In the absence of a physiologically induced system of blood loss such as menstruation, men’s creation of one, symbolically at least, is a male form of menstruation. While symbolic it is no less deeply cultural. For men this blood loss comes to signify the removal of contagion and facilitating in the creation of a boy into man. The ritual release is not only intended to remove bad or waste blood (*blut nogut; pipia blut*), but to create new, ‘good’ blood. As dirty or bad blood, it is imperative that men release this blood in order to maintain their health, strength, vitality, and their masculinity:

Okay, each man knows his body. If he is well, you will see that his body is strong, he can work, he can carry heavy things and when he has some kinds of sickness in his body in his blood system, you will see that he will not feel well. *Valentine, M, ESP*.

Now we grow to become healthy and it’s like you become a man now. *Aime, M, EHP*.

One informant from NCD also commented on the need to induce penile bloodletting in order to improve the look of a man’s skin; to make it beautiful:

Because women have a way of removing blood, they remove it through menstruation and you know, they get rid of it and their skin is bright all the time, and you know they become good flowers that go around and we desire. But we boys too, we are also flowers too and there is a way to make us become bright, and women will desire us. ^f^*Enoch, M, NCD*.

In addition to the references of maternal blood, health and masculinity one other reason for the need to release blood was offered in the contemporary context. Suggesting that the need to release blood is a regular, minor occurrence (‘It’s nothing’) Enoch from NCD, who originates from Madang, said that those men who have been ‘too sexually active’ must induce urethra bleeding every few weeks. From his narrative it appears that too much sex with women is linked with contagion in a way similar to maternal blood and therefore must be regularly released in order to prevent disease. This issue was also addressed by Valentine, a village elder from ESP:

It depends when he is sick. When he feels sick or has some kind of sickness in his body, alright he needs this sort of treatment [shooting]. He must go to this. *Valentine, M, ESP*.

One respondent who had heard of this tradition in his village in EHP reflected on the traditional transformation of penile shooting whereby, today young men were choosing to undergo dorsal slits for precisely the same reasons their ancestors had practiced penile and nasal bloodletting.

It’s like the practice of shooting the nose and such. Shoot a spear [arrow] at the head of the cock of the men, it is custom. It is a custom of the past which and still is now. Now we are here, it is a new time where similar customs are dying now and men are only going for the practice of cutting the foreskin. *Harold, M, EHP*.

Traditional penile shooting associated with ceremonial and cultural observances now appears to be rare, largely confined to *tambunataim* (the time of the ancestors):

I can’t tell you about this [penile shooting] because it is not done during our time. It was during our grandfather’s time and is already finished. Now, during our time, there isn’t any more of this practice. It ended during our father’s and grandfather’s time. *Hadrian, M, ESP*.

Although a few opinions were provided, it was unclear exactly why penile shooting in association with initiation had ceased in traditionally practicing areas. One elder spoke of the role of Christianity in bringing an end to penile shooting as it was deemed a ‘bad custom’. Valentine, another village elder in ESP, told us that penile shooting ceased in his village during the 1980s and that traditional knowledge associated with these practices have now largely been lost:

It’s finished now…it’s not really finished, it is there but we don’t have the elders here anymore who can do it. So we go to the other village’s style to do to prevent diseases. It is because the knowledge of penile shooting died with the ancestors…because the elders who have knowledge to do this thing have all died. They took away these songs with them. They no longer give to, transfer to, other people, elders and like will hold it [the knowledge] and when we grow up they give it to us. They don’t give these things unnecessarily, it’s against the law; forbidden. Like because we don’t know this songs, the magic words. We’re not sure now. *Valentine, M, ESP*.

Valentine did however believe that penile shooting would be revived with the repair of the village *haus tambaran* (men’s spirit house). While elders such as Valentine and Sailor believe that the traditional custom of penile shooting is dead, many young men in these communities continue to practice a contemporary form of penile shooting. One informant in particular, Xavier, had induced penile bleeding to treat an illness, a few days before our arrival in the village by cutting the head of his penis with glass.

Contemporary penile bleeding practices outside the traditional context of male initiation appear to be practiced in the Sepik, Eastern Highlands and Madang areas. Reflecting on the use of this practice to bring health, Franco, a university student in Port Moresby, NCD, reflected on his and his community’s practices:

Regarding the shooting of the skin, shooting the head of the cock… we use the bamboo skin… split a bamboo. The tip will be very sharp, so you will go and shoot its head [into the urethra] and blood will come down. So, there are people who know. It is like, lately, boys from the village are doing it. They did it and it has become part of their custom. *Franco, M, NCD*.

### Risk and the disposal of blood and excised skin

As a result of re-using non-sterile cutting equipment, penile cutting in a non-clinical setting poses a high risk for HIV cross infection between initiates. That said, there appeared to be a high level of awareness of the risk posed by re-using blades. Many of the men reported in their narratives of their cutting procedure that they were required by the cutter to bring their own blade. In settings where more than one male was being cut men spoke of ‘one blade, one man’. It thus appears, at least, that because of the knowledge of HIV being transmitted by sharing contaminated cutting equipment the actual process of cutting does not pose a high risk to the men in this study.

The participants in this study knew that if an infection (*susu*) were to develop in the penis then biomedical treatment was needed in order to prevent further complications. Many of the men who had undergone such cuts spoke of accessing antibiotics from a friend or a family member who works at a health care setting. No participants spoke of attending a health facility for treatment. One serious risk posed by penile cutting is delayed access to clinical care. One participant in the study had experienced excessive bleeding and a peer tied a rope around the base of his penis to stop the bleeding. As a result of prolonged restriction the young boy experiences ongoing erectile dysfunction.

The most common form of blood disposal in all types of penile cutting practices involved allowing blood to flow into running water, mostly a river, creek or sea.

After the cut we stay in the water; the water washes the blood away. And when we come out of the water and we see blood still coming out, we continue to stay in the water for it to wash away the blood. And if not we sit on the stone and get the water and wash and clean the blood. *Cornelius, SC, EHP*.

Others said they let the blood flow into a reservoir of water such a dish which is later thrown away, while some collected the blood using leaves and/or pieces of cloth which were then burnt or buried.

In regards to the blood, it is in the house so we put a dish of water. When the blood comes down, we get water and put it on top of the what [penis]. After cutting we put this [blood] in the water and throw it away under house. *Lekemi, M, NCD*.

Respondents in ESP, EHP and NCD typically regarded blood simply as waste and of no future significance. However, some men thought of blood as having certain values, and therefore did not dispose of it but collected it for use afterwards. One study participant stated that blood from penile shooting was left to flow onto *tanugt* leaves that initiates then used to wash with in order to make them strong and healthy. *Tangut* leaves are leaves that are used in the garden to designate land boundaries and used in ceremonial activities to decorate the bodies of men and women.

Shoot its head [the penis] and get rid of this blood to come down onto the *tangut* [leaves] and then they wash. They wash with this *tangut* and get rid of it [the blood]. *Femson, M, ESP*.

It’s like, the oil [from the blood] you put on your skin and rub on your body, it will make your skin look healthy and when you walk around it can help and such. *Sailor, M, ESP*

Similarly, another interviewee said that the ‘oil’ from the blood collected following contemporary penile cutting practices was used to attract women.

When the blood comes down, I tell them, that, “this blood of yours that comes down, you get it. Do not let it go to waste. You have to get this blood. You put it in a bottle and you take it and you go put it in the sun. Put it in the sun and you will see that it will become almost like oil. This oil of yours, you can use on women or such… He tried it like I told him and women came directly to him. He came and told me, “I used that thing and it worked out”. *Henriot, M, ESP.*

Another interviewee stated that the blood from penile cutting was mixed with coconut flesh chunks and given to the initiates to eat and to rub on their bodies, again, to provide strength to initiates who do this.

Okay, the first blood that comes will shoot out with force like water from the water tap. The second one that come out, they get it and break the coconuts and rub it in the blood and then rub it on their skin and give to them to eat to strengthen them. *Sailor, M, ESP.*

It [the blood] doesn’t mean anything. It’s waste. *Bolton, M, NCD.*

In WNB, respondents said that blood that is released during traditional penile cutting ceremonies must be buried and ancestral spirits informed of this action and asked to protect the boys from developing complications:

Yeah, they take it and go bury it and tell our ancestors’ spirits that this is the bad blood we are burying. It has come from the bodies of our young boys and we are burying it and it will go forever. They talk to the spirits and the spirits will witness that they have buried this thing. *Chief, M, WNB.*

In addition, the burial of blood and remnants of foreskin in WNB are believed to contribute to the production of masculine identity of belonging to the clan and of now being an owner of the land.

## Discussion

The socio-cultural meanings underpinning many of these practices appeared to overlap, with evidence of appropriation observed between traditional (customary) and contemporary penile practices thus contributing to the conditions for contemporary practices. As such there was change within continuity and continuity within change. Constructions of masculinity, men and men’s constructions of women’s sexuality and a desire for enhanced personal and partner sexual pleasure appeared to be strong motivators for both contemporary penile modification practices, with evidence that some men seek progressive penile modification (e.g. contemporary, longitudinal dorsal slit of the foreskin followed a variable time later by the insertion of ball bearing(s) into the upper skin of the remaining foreskin adjacent to the upper surface of the glans). Contemporary cutting appeared to be the most common penile practice, the key motivator for which was to increase sexual pleasure for men and their female partners, and to improve genital hygiene and sexual health*.* In contemporary cutting, the most common cut was the ‘straight cut’, preferred because the foreskin is not removed but left to hang down under the penis and which is believed to result in increased sexual pleasure for both partners (Figure 2a).

This qualitative description of current penile practices, the settings in which they take place and the socio-cultural meaning assigned to them is one of the most comprehensive descriptions of these practices in PNG in recent years. Despite rapid modernisation and missionisation, there are cultural groups in PNG who continue practicing customary rituals associated with the creation of men and masculine identity through penile modification. While customary penile cutting was not the most dominant form of current penile modification in this study it was far more apparent than is currently evident in a variety of HIV behavioural research.^g^ For example of the 128 male respondents attending an STI clinic who reported being circumcised none reported that they were cut during a traditional initiation [27]. This is likely to be a positive sampling issue whereby this study was conducted in a diversity of areas and not contained to the Highlands and Port Moresby but rather included the Islands region where this practice is common. Contemporary penile cuts appear to be those of greatest diversity in terms of both the type of cuts practiced and in terms of the presence of this cultural context of penile modification in all regions of the country. Despite the differing socio-cultural locations in which these practices are occurring, notions of masculinity are central to all penile practices irrespective of whether the emphasis is on sexual pleasure, health or strength. That said notions of male and female sexuality appear to be far more strongly associated with contemporary penile cuts than with tradition ones which emphasise masculinity rather than sexuality.

Like all forms of customary practices, traditional penile cutting practices are also subject to change. This was well evidenced by Tuzin [34] in his follow up book on the death of the haus tambaran [men’s spirit house] and by association the demise of masculinity in the community where he once vividly described male cult practices and violent penile bloodletting practices. In this study we see the appropriation of modern medical equipment in the use and treatment of traditional penile cuts, including in a few instances the removal of the penile cut from the community setting into the medical environment. Furthermore, the types of food shared at the feast have evolved with modernisation and the introduction of rice and money to purchase foods that would have otherwise been grown. In addition, the role of cash in the compensation of the cutter and to the mother’s brothers also highlights that the customary practice of penile cutting is also open to and subjected to influences of modernity and medical practice. Other forms of cultural modification were evident with men from non penile bloodletting cultures now undergoing this sometimes radical penile transformation with others still now inserting objects into their penises after exposure to this practice from elsewhere.

The genesis for the larger study from which this paper is drawn came from a growing discourse within PNG addressing the feasibility and appropriateness of MC as a biomedical HIV prevention method. Aggleton [13] argues that ‘male circumcision must be promoted in a culturally appropriate, rights-based and gender sensitive way’. In the context of PNG, this does not simply imply sensitivity towards, and an understanding of, the context and meanings associated with traditional penile practices for contemporary practices are also deeply cultural and imbued with meaning and it appears that many of the reasons for the use may be important in any future consideration of medical circumcision. Any discussion of implementing male circumcision for the prevention of HIV in PNG would need to address the significance and context of both traditional and contemporary practices, since both embody meanings far more complex and rich in metaphor than suggested simply by the type of cut, insert or other procedure carried out. As the title of this paper applies penile cutting practices in PNG is more than just a cut.

There are some penile practices that are not thoroughly addressed in this paper and they include the injection of substances into the penis. While the injection of substances was mentioned in ESP and the application of herbs and liquids were mentioned, no one in the study had actually been involved in these practices. This is in contrast to the work of others [28]. Furthermore, except that the injection would cause an increase in penile size, a larger penis in and of itself does not pose a greater risk for HIV transmission. The injecting, if done unsafely does. This paper had a somewhat different focus because it forms part of a larger in-depth qualitative understanding of penile practices in the context of male circumcision for the prevention of HIV.

## Conclusions

This study has highlighted that there has been a translocation of penile modification in PNG from the traditional and customary to the contemporary and more recently the clinical setting. The implication of this cultural transition is both theoretically important but also critically important to discourses and practices around medical male circumcision for the prevention of HIV; in particular the transition from an emphasis on masculinity to an emphasis on sexuality and hygiene which may place men at greater risk. Any penile modification, as this paper has highlighted, whether it is a cut or an insert is deeply cultural and imbued with social meaning. In this way, such modifications are as Aggleton [13] said ‘far from a simple technological act’ (p.15) and more than just a cut. These practices are imbued with meanings and social significance about blood, kinship, masculinity, health and illness. There are also, at least in the traditional setting, linked to the spiritual. While by themselves these practices and their meanings are important, when read in the context of public health, particularly HIV prevention and recent pushes to scale up biomedical prevention through the roll out of male circumcision programs, their importance takes on greater resonance. For example, Kippax [47] has argued that HIV prevention information is rarely accepted without appropriation and how it is appropriated is dependent upon the social and cultural contexts of the milieu in which it is being provided. Any introduction of a new penile practice (male circumcision as an HIV prevention technology) will undoubtedly alter the culture of current practices in PNG and this alteration must be documented and assessed in terms of maintaining cultural integrity including sensitivity to customary practices and the creation of men and in its impact on gender and sexual relationships. Moreover, any public health program that would seek to implement adult male circumcision would have to address the current state of penile modification in PNG, including the beliefs that men have regarding their protection from HIV and other STIs as a result of their current penile cut and the ambiguity that exists between different forms of penile cuts and that associated with MC. Without understanding in detail current penile practices it is impossible to effectively communicate about MC, including its difference from other cuts, and the benefits and risks associated with MC within the context of prevailing penile cutting and modification practices.

In order to address the public health concerns that these practices raise, a new phase of HIV prevention in PNG is required which draws upon principles of harm reduction. It may not be possible, or desirable in some situations, to bring an end to penile modification practices. Attention and efforts may be better targeted at reducing the risk of HIV transmission between initiates and between initiates and cutters. In addition harm reduction in this context must address the prevention of complications such as haemorrhage, infection and erectile dysfunction for example. Therefore an integration approach to harm reduction is required that combines public information and education campaigns, health provider training, provision of clinical services to men suffering complications from non-medical forms of penile cutting, and support for safer cutting procedures outside the formal health sector. Further attention must also be placed on supporting peer education whereby the risks associated with the sexual transmission of HIV post penile cuts are explored and understood. Moreover, in the context of widespread longitudinal cuts where the foreskin remains further research is required to understand what, if any, protection longitudinal cuts may provide to men against HIV. Ultimately, to overlook the current penile practices, their meanings and their public health risks would have a disastrous impact on any intervention program that advocates for MC. In addition to taking account of women and men’s acceptability of MC, to ignore the current cultural context of penile practices may undermine any clinical benefit of MC. In fact, the cultural meanings of contemporary non-customary forms of penile cutting and bloodletting may provide avenues to promote MC in PNG, if deemed an appropriate technology to stem the epidemic.

### Endnotes

^a^ In one community, the village chief advised initiated men in the community not to share important spiritual aspects of male initiation but did approve the discussion of previously held secret knowledge with male members of the research team. In order to acknowledge the sharing of secrets that could potentially upset their spiritual ancestors, a financial contribution from the team was made. This money was used for a feast in which the men would sing, share food and call upon their ancestral spirits to give thanks for their knowledge and to acknowledge the sharing of secret information. In addition, the Chief made an offering of a traditional bride price necklace to the first author in recognition of the sharing of customary knowledge and practices and the research team’s respect of custom.

^b^ We use the term ‘cutting’ but the authors of this paper are conscious of the limitations of its use in the traditional context and are aware of its neutrality and that it appears insensitive to the pain undergone by the novice where there is a “dramatically staged assault on the penes of the novices” (p.66) 21. Tuzin DF: *The Voice of the Tambaran: Truth and Illusion in IlahitaArapesh Religion*. Berkeley: University of California Press; 1980.

^c^ One of the informants went into great detail about the stages of initiation and while the traditional cut is undertaken at the first stage when a boy is aged around 7 years he is then expected to go through another initiation to make him an adult and it is during this stage that his clan’s spirit will whip him and he will seek protection from his uncles.

^d^ The tokpisin word ‘smel’ was used and in the context of this quote the smell is not so much the smell of polluting substances such as menses but rather refers to the bodily smell of the women which is not polluting but powerful and would then disrupt the magic which the male elders had already performed.

^e^ Although this respondent was talking about contemporary penile cutting his reference here to ‘bad blood’ is associated with traditional notions of bleeding. There appeared to be an appropriation of traditional beliefs regarding blood in the contemporary context.

^f^ One of the health care workers in Kimbe, West New Britain recalled a woman whom she cared for who had a rubber band in her uterus and which only came out after delivering her child. It appeared from this health care worker that men are also putting rubber bands on their penises.

^g^ This reference to skin reflects the important role that skin plays in Melanesian society. For example amongst the Daribi people bad skin comes to reflect overindulgence in sex and therefore exposure to polluting female substances. In this context skin becomes a symbol of a moral state and not simply a bodily sign. See [48]. Strathern M: *Body Thoughts*. Ann Arbor: The University of Michigan Press; 1996.

## Abbreviations

MC: Male circumcision; PNG: Papua New Guinea; HIV: Human immunodeficiency virus; WNBP: West new britain province; ESP: East sepik province; NCD: National capital district; EHP: Eastern highlands province.

## Competing interests

The authors declare that they have no competing interests to report.

## Authors' contributions

AV, JK & PS conceived the Male Circumcision Acceptability and Impact Study (MCAIS) which this is part of. AK, AV & LF designed the qualitative component of the MCAIS. AK & AV oversaw data collection. AK, MK, JN & RN collected data and AK, MK, JN, RN, HA & AV conducted all data analysis. AK & AV lead the writing of the article. JK & LF reviewed early drafts and provided feedback. All authors read and approved the final manuscript.

## Pre-publication history

The pre-publication history for this paper can be accessed here:

http://www.biomedcentral.com/1472-698X/12/10/prepub
